# Surgical Experiences: The Septic Infection of Wounds

**Published:** 1915-04-10

**Authors:** 


### SURGICAL EXPERIENCES.

### The Septic Infection of Wounds.

### SIR ALMROTH WRIGHT'S LECTURE AND CONCLUSIONS.

On Tuesday, March 30, Sir Almroth Wright, F.R.S., delivered a lecture, in the Robert Barnes Hall of the Royal Society of Medicine, to a very large audience, on "The Septic Infection of Wounds." The chair was occupied by the president, Dr. Frederick Taylor. The following is a condensed report of his remarks:—

#### The Conditions at the Front.

The lecturer first alluded to the conditions obtaining at the Front, and the consequent infection of wounds, declaring that there was not a case of which that statement was not true, especially as the projectile carried deep into the tissues infected clothing and other material from without. Following on the infliction of the wound there was an effusion into the wound of both blood and lymph, which constituted a suitable culture medium for microbes. He limited his attention to the three chief microbes which had been found—the streptococcus, the *Bacillus ærogenes capsulatus* of Welch (or gas phlegmon of the Germans), and the tetanus bacillus. Of these the one which was practically ubiquitous was the streptococcus; it was the most dangerous because it multiplied rapidly in the blood itself and needed no pre-digestion of the pabulum in which it found itself in order to establish colonies. He believed the end of these cases of pathogenic infection would not be seen for years. We were now "up against" the biggest problem in bacterial infection which the world had ever seen, and it became of the utmost importance to determine what was really the best treatment of these cases, of which this war had probably produced a couple of millions. The treatments at the present time consisted of (1) antiseptic treatment; (2) a series of procedures which he grouped as physiological methods—*i.e.* those which brought the natural anti-bacterial power of the blood to bear; (3) inoculation (increasing the anti-bacterial power of the blood by vaccine therapy).

#### Antiseptics and Inoculation.

The antiseptic method he regarded as merely ancillary to that of using the physiological processes, as, indeed, was the system of inoculation. Antiseptics could not be brought into contact with every bit of disease-bearing surface. The inquirer was, therefore, inevitably driven to study what takes place in the wound itself; only in that way could be evolved an efficacious system of treatment. The only methods which applied were the laboratory methods, with pus and undiluted blood. He proceeded to demonstrate a method by which he ascertained the materials and conditions which facilitated the growth of microbes, a very important factor being the importation into the wound of trypsin. In some cases a light sowing of microbes produced no result, but a heavy sowing of the same variety caused the formation of colonies; they seemed to work in co-operation in reducing the anti-tryptic power of the blood—a necessary condition to their multiplication. Particular doses of some antiseptics stimulated the growth of microbes, instead of killing them. The usual attitude with regard to antiseptics was: antiseptics killed microbes, and nothing else was known which directly did that, and even if all the microbes in a wound were not killed by the treatment, most of them were, and so something was gained.

#### The Limitation of Vaccines.

But the important fact was that the method had not sufficient penetrative power to kill all. Complete sterilisation of a wound must be given up as a dream which would never be realised. And the faith which led a man to say it was a gain to kill some, if not all, could not have had origin in a laboratory; for whether one microbe or a million were left in a test-tube, by next day there would be prolific numbers in it, provided only the culture medium were good. He believed that every surgeon expected a similar multiplication in his wounds. The essential requirement was to find something which would prevent the corruption of the lymph; unless the nutrient medium were so changed that microbes would not grow, the killing off of some of the microbes would continue to be more or less futile. It had been found of the greatest service to stimulate the free flow of lymph into the wound by the employment of lymphagogues, such as a solution of 5 per cent. salt and ½ per cent. citrate, the latter being added to prevent clotting. It was essential to realise that vaccines had their limitations, for the protective substances formed as a result of this measure did not penetrate everywhere. It was not expected to be of very great service for an unopened abscess, or a badly drained wound. But a cellulitis had often been immediately checked by giving a vaccine.

He concluded by an eloquent reference to what he termed "the higher policy" of this question. We were faced now with a situation in which we had to deal with millions of wounds, and opinion was very varied as to what ought to be done, whilst very little was being done except on the old lines. The medical profession was very democratic, in the sense that every man before adopting a line of treatment insisted that it must recommend itself to his mind, which was an admirable attitude in circumstances where he was constantly in touch with his cases and saw the results from time to time.

#### Instructions from Headquarters.

But in the present war a doctor saw a case for a day or two, and it was then transferred somewhere else; he did not know how his colleague at the Front had treated the patient in the first place, and there was similar lack of knowledge as to prior treatment in the case of each surgeon. To guide oneself by tradition and the advice of the next man was the wrong method in medicine. The practically compulsory inoculations against typhoid and tetanus had produced marvellous results; and that showed how important, in this condition of affairs, was a series of instructions from headquarters as to what treatments should be carried out. At present, practice was very varied, and it was urgently desirable that the powers that be should say "You must no longer do as you like." He thought the time had come when the whole profession should think out what should be done. The material was not yet complete enough to enable the law to be laid down: but just as the world at this juncture is looking to Britain to provide munitions of war, so it is looking to her to lead in research in medicine.

